# Enzymatic Hydrolysis and Characterization of Protein Concentrate Obtained From Mechanically Separated Meat of Nile Tilapia (*Oreochromis niloticus*)

**DOI:** 10.1111/1750-3841.70799

**Published:** 2025-12-26

**Authors:** Poliana dos Santos Mendes, Flávia Aparecida Reitz Cardoso, Maria Fernanda Giugiolli, Gabriela Wolhmuth, Maria Isabel Campos Oliveira, Vitória de Freitas Fante, Jéssica Novelo Nascimento Brenag, Evandro Bona, Patrícia Casarin, Adriana Aparecida Droval Arcain

**Affiliations:** ^1^ Postgraduate Program of Food Technology (PPGTA) Federal University of Technology–Paraná (UTFPR) Campo Mourão Brazil; ^2^ Postgraduate Program of Technological Innovations (PPGIT) Federal University of Technology–Paraná Campo Mourão Brazil; ^3^ Department of Food Engineering and Chemical Engineering Federal University of Technology–Paraná (UTFPR) Campo Mourão Brazil

**Keywords:** alcalase, degree of hydrolysis, emulsifying properties, fish protein hydrolysate, mechanically separated meat, papain

## Abstract

The valorization of mechanically separated meat (MSM) from Nile tilapia through enzymatic hydrolysis can convert an underutilized co‐product into functional ingredients; however, a practical balance between the degree of hydrolysis (DH) and techno‐functional performance remains underexplored. This study aimed to optimize hydrolysis conditions and clearly define the treatments, enzyme (alcalase or papain), concentration (0.3975%–1.1025%), and time (0.385%–4.615 h), and to characterize the resulting fish protein hydrolysates (FPHs) in terms of solubility and emulsifying properties. A central composite rotatable design was used to evaluate the specified factors. Responses included solubility at pH 5.0, 7.0, and 9.0, emulsifying activity index (EAI), emulsion stability index (ESI), and qualitative FTIR. The optimized condition with alcalase (1.1025%, 4.615 h) yielded a DH of 13.80%, while the design‐center condition yielded 2.06%, accompanied by improved solubility at neutral/alkaline pH and higher EAI/ESI. Papain tended to generate higher DH ranges in certain conditions but exhibited lower solubility near its isoelectric point (pI). Principal component analysis captured enzyme‐dependent patterns linking DH and solubility. Collectively, the results indicate that tuning enzyme specificity, dose, and time enables the production of tailored FPH with balanced functionality, making it potentially suitable for use as clean‐label emulsifiers or for protein enrichment, thereby supporting circular economy strategies by upcycling MSM. Because in‐depth structural assays (e.g., peptide profiling, amino acid composition, zeta potential) were beyond the scope, we identify them as future steps to strengthen structure–function interpretation and validation in real food matrices. Standardized reporting of units (% DH) and clear treatment definitions can facilitate comparability across studies and accelerate the translation of findings.

## 1 Introduction

Protein‐rich foods of aquatic origin have gained increasing prominence in the global food landscape due to their high nutritional value and socio‐economic relevance. Several studies have highlighted that fish provide high‐quality proteins with all essential amino acids, as well as polyunsaturated fatty acids, vitamins, and important minerals for human health (Sila and Bougatef 2016; Siqueira 2017). The expansion of aquaculture, combined with growing consumer demand for healthy and sustainable food, has contributed to the rising consumption of fish and fish‐based products. Among freshwater species, the Nile tilapia (*Oreochromis niloticus*) stands out for its favorable traits, including rapid growth, high carcass yield, easy reproduction, and excellent sensory characteristics of the meat (Romanzini 2023). In industrial processing, mechanically separated meat (MSM) is produced by mechanically grinding and separating the fish frame, a procedure that removes bones and spines, yielding a homogeneous, boneless mass rich in myofibrillar proteins (i.e., free of bone fragments).

In Brazil, tilapia accounts for more than 65% of total aquaculture production, with the state of Paraná leading with over 200,000 tons per year (Peixe BR 2024). However, tilapia processing, particularly filleting, generates a significant amount of organic solid waste, estimated at 60%–72% of the total fish biomass (Matiucci, Lima, et al. 2021; Zamora‐Sillero et al. 2018). These by‐products include heads, skins, viscera, scales, bones, and especially MSM. In this process, the meat is obtained through a mechanical grinding process, resulting in the separation of bones and spines. MSM is rich in myofibrillar proteins and, despite its nutritional and functional potential, is often underutilized in the food industry, commonly discarded, or directed to animal feed (Borgogno et al. 2017). Beyond MSM, other fish by‐products (e.g., scales) have been valorized via enzymatic hydrolysis and membrane fractionation to obtain peptide‐rich fractions (Zu et al. 2023), reinforcing the technological scope for upcycling diverse streams.

In light of growing concerns about food waste and the search for recovery strategies, the valorization of fish by‐products has become a promising alternative to promote sustainability and the circular economy in the aquaculture production chain (Decker et al. 2016; Khiari et al. 2022). In this context, enzymatic hydrolysis emerges as a viable and effective technology to convert complex proteins into peptides with improved techno‐functional properties and, in many cases, specific biological activities (Chalamaiah et al. 2010). The use of specific proteolytic enzymes enables the modification of protein structure, increasing solubility, bioactivity, and functional performance, with potential application in processed foods, beverages, supplements, and clean‐label formulations (Oliveira et al. 2017; Vo et al. 2020). Recent studies have demonstrated that fish protein hydrolysates (FPHs) can be successfully incorporated into emulsified meat products, high‐protein beverages, and nutritional supplements, providing both functional and health‐promoting effects. The relationship between secondary structures and functionality has also been highlighted in other matrices, where spectroscopic markers inform techno‐functional behavior (Singh et al. 2023), supporting the rationale for qualitative Fourier transform infrared (FTIR) context in protein hydrolysis studies.

Among the most widely used enzymes in the hydrolysis of animal proteins are alcalase, a serine endopeptidase derived from *Bacillus licheniformis*, and papain, a cysteine protease extracted from papaya latex (*Carica papaya*). Alcalase acts preferentially on peptide bonds adjacent to hydrophobic amino acids, producing smaller and more uniform peptides. Papain, in contrast, has broader specificity, with endopeptidase, amidase, and esterase activity, acting on hydrophobic and aromatic residues (Tavano 2013). These enzymatic differences result in hydrolysates with distinct peptide profiles, which directly impact properties such as solubility, emulsion stability, and emulsifying activity (Kumari, Kaushik, et al. 2023). Emerging applications across alternative protein systems suggest a broader potential for peptide hydrolysates in food and health contexts (Park et al. 2023), thereby expanding the relevance of functional optimization beyond a single matrix.

Although enzymatic hydrolysis of tilapia by‐products has been studied in various contexts, most research focuses on whole or mixed by‐products (e.g., heads and viscera) or aims primarily at generating bioactive peptides. Few studies explore a comparative evaluation of commercial enzymes applied specifically to tilapia MSM, a by‐product with a unique composition, under optimized functional conditions. Furthermore, many studies overlook important experimental variables such as enzyme concentration, hydrolysis time, and pH, which can significantly affect the quality and functionality of hydrolysates (Shu et al. 2018). In this regard, the present study addresses the need for systematic, multivariate optimization focused on techno‐functional outcomes in tilapia MSM while recognizing that deeper structural analytics are valuable but beyond the current scope.

Another important aspect concerns the structure of the resulting peptides. The relationship between structure and functionality is well established, especially in relation to solubility and emulsion stability. However, more specific structural analyses, such as peptide size distribution (e.g., SDS‐PAGE, chromatography), amino acid composition, or zeta potential, are rarely performed in studies involving tilapia by‐products, mainly due to cost, time, or analytical limitations. The absence of these evaluations makes it difficult to understand, at a mechanistic level, why certain hydrolysates, such as those produced with papain, may exhibit high DHs yet poor solubility. Here, we explicitly delimit the scope to techno‐functional optimization and comparative enzyme evaluation; deep structural analyses are acknowledged as a limitation and are identified as future work to refine structure–function interpretation.

In this scenario, the application of statistical optimization tools, such as the central composite rotational design (CCRD), can significantly contribute to defining optimal hydrolysis conditions based on the desired functional outcomes. Within a response surface methodology (RSM) framework, this approach enables the simultaneous exploration of multiple variables and identifies the optimal combinations of enzyme concentration and hydrolysis time to maximize responses, such as solubility, emulsifying activity index (EAI), and emulsion stability index (ESI). Moreover, spectroscopic analyses such as FTIR spectroscopy can provide qualitative evidence of structural modifications in proteins following enzymatic hydrolysis, even in the absence of peptide quantification (Barth 2007).

Therefore, the present study aimed to obtain a protein concentrate from Nile tilapia MSM and evaluate its enzymatic hydrolysis using alcalase and papain. Different enzyme concentrations and hydrolysis times were tested and optimized using a central composite rotational design. The functional properties of the hydrolysates, including solubility, EAI, and ESI, were evaluated, and FTIR analysis was conducted to investigate structural changes. The originality of this work lies in seeking a balance between the degree of hydrolysis (DH) and functional properties rather than simply maximizing DH, aiming to produce hydrolysates suitable for incorporation into diverse food products. While deep structural analyses (peptide profile, zeta potential, and amino acid composition) were not included in the present scope, they are recognized as a limitation and are designated as future steps to strengthen the understanding of structure–function relationships. Accordingly, the gap addressed here concerns the lack of systematic, application‐oriented optimization for tilapia MSM using multivariate design and coherent functional endpoints.

## 2 Methodology

The study was conducted in the laboratories of the Federal University of Technology—Paraná (UTFPR, Campo Mourão Campus) within the Graduate Program in Food Technology (PPGTA). UTFPR's multiuser laboratory facilities were also utilized.

### 2.1 Raw Material

A fillet processing company in the western region of Paraná state kindly provided the MSM from Nile tilapia. Upon receiving the frozen samples, they were stored in a freezer at −18°C. MSM was obtained by mechanical grinding and separation of the fish frame, a process that removes bones and spines, yielding a homogeneous, boneless mass rich in myofibrillar proteins (i.e., free of bone fragments).

The enzymes used were alcalase 2.4 L, a serine endopeptidase obtained from *B. licheniformis* (≥2.4 AU g^−1^; liquid; Sigma‐Aldrich, St. Louis, MO, USA), and papain, a cysteine protease from papaya latex (*C. papaya*) (≥3.5 U mg^−1^ protein; powder; Sigma‐Aldrich, St. Louis, MO, USA). Both enzymes were purchased with financial support from UTFPR research funding programs.

### 2.2 Preparation of Protein Concentrate

The protein concentrate was obtained following the methods of Eisele and Brekke (1981) and Vidal et al. (2011), with modifications made in this study. MSM samples were partially thawed in a refrigerator at 4°C for approximately 8 h before analysis. Subsequently, 100 g of MSM was weighed and washed at a 1:3 (w v^−1^) (solid:liquid) ratio.

The first wash was performed with refrigerated distilled water (Type II, resistivity ∼1 MΩ cm^−1^) at approximately 10°C, using an inverted water bath (model Q215M2, Quimis, Diadema, Brazil), and mechanically agitated using a benchtop mechanical stirrer (model 713, Fisaton, São Paulo, Brazil) at 1500 rpm for 5 min. The sample was filtered using a polyester microfiber bag (pore size 50 µm, Cetromaq, São Paulo, Brazil) to remove excess water. The washing liquid was discarded after filtration.

The second wash was performed with 0.05% (v v^−1^) phosphoric acid (H_3_PO_4_, ≥85%, analytical grade, Sigma‐Aldrich, St. Louis, MO, USA), mechanically agitated for 15 min at ∼10°C under the same mechanical stirring conditions. This aimed to deodorize the protein concentrate and adjust the pH to ∼5.0 (the isoelectric point [pI] of myofibrillar proteins in meat, as described by Kristinsson and Hultin 2003). At the isoelectric point, proteins exhibit the lowest solubility, forming precipitates. After allowing the MSM solids to settle, filtration was performed.

The third wash was done with distilled water under the same conditions as the first. After a settling period of approximately 5 min, the sample was filtered again. The washing liquid was discarded, and the precipitated solid portion from the final MSM wash was placed on aluminum foil‐lined trays and dried in an air circulation oven (model CE‐150/3, Cienlab, Campinas, Brazil) for 15 h at 50°C.

The dried material underwent a wash with 70% (v v^−1^) ethanol (≥99.5%, analytical grade, Merck, Darmstadt, Germany) to reduce lipid content, was filtered through a 75‐mm‐diameter stainless‐steel sieve (mesh size 200, 75 µm, Bertel, Caieiras, Brazil), and was dried again in the oven for 3 h at 50°C. The dried protein concentrate was ground in a blender (model RI2137, Philips Walita, Varginha, Brazil), packed in polyethylene plastic bags, and stored in a vertical freezer (model H500, Metalfrio, São Paulo, Brazil) at −18°C for further analysis. All analyses were performed in triplicate (*n* = 3).

### 2.3 Proximate Composition Analysis of the Protein Concentrate

The gravimetric method was used to determine the moisture content at 105°C in a drying oven (model CE‐150/3, Cienlab, Campinas, Brazil) until a constant weight was achieved. Protein content was determined using the micro‐Kjeldahl digestion process in a semiautomatic Kjeldahl system (model TE‐036/1, Tecnal, Piracicaba, Brazil), with a nitrogen‐to‐protein conversion factor of 6.25. Both methods followed the *Physico‐Chemical Methods for Food Analysis* (Instituto Adolfo Lutz 2008). Lipid content was determined according to Bligh and Dyer (1959), using cold extraction with a mixture of chloroform (≥99.8%, analytical grade, Sigma‐Aldrich), methanol (≥99.9%, analytical grade, Sigma‐Aldrich), and distilled water (Type II, resistivity ∼1 MΩ cm^−1^).

### 2.4 Hydrolysis Process

Enzymatic hydrolysis was carried out using alcalase 2.4 L (*B. licheniformis*) and papain (*C. papaya*) (both from Sigma‐Aldrich, St. Louis, MO, USA), under their respective optimal conditions (pH 8.0, 54°C for alcalase; pH 5.5, 50°C for papain), as previously described in Section 2.1.

To evaluate the influence of enzyme concentration and hydrolysis time on the protein concentrate, a central composite rotatable design (CCRD) was employed (Table 1). The CCRD was designed to determine the optimal hydrolysis conditions and obtain a complete quadratic model describing the enzymatic process. The design included two central points (for lack‐of‐fit testing) and two axial points (*α* = ±1.41).

The enzymes were added to the protein concentrate at concentrations of 0.50%, 0.75%, and 1.00% (w/w, relative to protein), and incubated for 1.0, 2.5, and 4.0 h. The dependent variables evaluated were the DH (%), solubility (%), EAI (m^2^ g^−1^), and ESI (min).

For each experiment, 10 g of substrate (protein concentrate) was weighed, and 50 mL of distilled water (Milli‐Q, Merck Millipore, Darmstadt, Germany) was added. The pH was adjusted with 0.05 M NaOH and/or 0.1 M HCl until the optimal pH for each enzyme was achieved. The slurries were maintained under orbital shaking (120 rpm) in a thermostatic water bath (TE‐184, Tecnal, Piracicaba, Brazil).

*TCA precipitation*: Samples were treated with 6.25% trichloroacetic acid (TCA) (Synth), allowed to stand for ∼10 min, and centrifuged (model 5804 R, Eppendorf, Hamburg, Germany) at 6000 rpm for 20 min at 4°C. After centrifugation, the supernatant was filtered through Whatman No. 40 filter paper. The filtrate was used to determine TCA‐soluble nitrogen for DH calculation.

*Thermal inactivation*: The remaining solution was heated at 90°C for 15 min. The inactivated sample was then lyophilized (model L101, Liotop, São Carlos, Brazil) for subsequent analyses of solubility, EAI/ESI, and FTIR spectroscopy (IRPrestige‐21, Shimadzu, Kyoto, Japan; ATR‐diamond).

### 2.5 Determination of the Degree of Hydrolysis

The DH (%) was determined according to the methods of Hoyle and Merritt (1994) and Baek and Cadwallader (1995), with minor modifications. After hydrolysis, an aliquot of each reaction was centrifuged at 6000 rpm for 20 min at 4°C, and the supernatant was collected to determine soluble proteins. The protein content of the soluble fraction was determined by the biuret method, whereas the total protein content of the original protein concentrate was measured by the micro‐Kjeldahl method (conversion factor 6.25).

DH was calculated using Equation (1), which expresses the percentage of soluble proteins relative to the total protein in the sample.

(1) $$\mathrm{DH}\;\;\left(\%\right)=\frac{\mathrm{Soluble}\;\mathrm{proteins}}{\mathrm{Total}\;\mathrm{protein}\;\mathrm{of}\;\mathrm{the}\;\mathrm{sample}}\times \;100$$

### 2.6 Determination of Solubility of Fish Protein Hydrolysates

Protein solubility was determined according to Chalamaiah et al. (2010), with minor modifications. Briefly, 300 mg of FPH was diluted in 30 mL of distilled water (Milli‐Q, Merck Millipore), and its solubility was assessed at pH levels of 5.0, 7.0, and 9.0. The pH was adjusted using 0.5 N HCl and/or 0.5 N NaOH. The solutions were stirred at 25 ± 2°C for 30 min and centrifuged at 6000 rpm, 30 min, 4°C in a refrigerated centrifuge (model 5804 R, Eppendorf, Hamburg, Germany). Protein in the supernatant was determined by the biuret method, and total protein was determined by the micro‐Kjeldahl method (IAL 2008). Absorbance readings (biuret) were performed at 550 nm using a UV‐Vis spectrophotometer (model UV‐1800, Shimadzu, Kyoto, Japan).

(2) $$\mathrm{Solubility}\;\;\left(\%\right)=\frac{\mathrm{Protein}\;\mathrm{in}\;\mathrm{the}\;\mathrm{supernatant}}{\mathrm{Total}\;\mathrm{protein}\;\mathrm{in}\;\mathrm{the}\;\mathrm{sample}}\times \;100$$

### 2.7 Emulsifying Capacity of Fish Protein Hydrolysates

With some modifications, the emulsifying capacities were determined according to the method described by Pearce and Kinsella (1978). The emulsifying capacity was measured at pH 5.0, 7.0, and 9.0. Hydrolyzed fish protein (HFP) samples were prepared at a concentration of 1.0 mg mL^−1^ in distilled water (Milli‐Q, Merck Millipore, Darmstadt, Germany). A 90 mL aliquot was taken, and the pH was adjusted to 5.0, 7.0, or 9.0 using 0.5 N sodium hydroxide (NaOH, analytical grade, Synth, Diadema, Brazil) and/or 0.5 N hydrochloric acid (HCl, analytical grade, Synth). After pH adjustment, 30 mL of soybean oil (Soya, Bunge Alimentos, Gaspar, Brazil) was added to each mixture.

Each mixture was homogenized using an Ultra‐Turrax T18 basic homogenizer (IKA, Staufen, Germany) at 20,000 rpm for 1 min. Then, 100 µL of the emulsion was pipetted from the bottom of the mixture and diluted in 10 mL of 0.1% (w v^−1^) sodium dodecyl sulfate (SDS) solution (≥ 99% purity, Sigma‐Aldrich, St. Louis, MO, USA) at 0 and 10 min after homogenization. Absorbance values were measured at 500 nm using a UV‐Vis spectrophotometer (UV‐1800, Shimadzu, Kyoto, Japan).

The absorbance values at times *A*_0_ and *A*_10_ were used to calculate the EAI (Equation 3) and ESI (Equation 4).

(3) $$\mathrm{EAI}\;\;\left(m^{2}g^{-1}\right)=\frac{2\;\times \;2.303\;\times \;\mathrm{DF}\;\times \;A}{C\;\times \;0.25\;\times \;1000}$$

(4) $$\mathrm{ESI}\;\;\left(\min\right)=\frac{A_{0}}{A_{0}-\;A_{10}}\times \;10$$

where DF is the dilution factor (100), C is the initial hydrolysate concentration (g mL^−1^), *φ* = 0.25 is the oil volume fraction, and *A*_0_ and *A*_10_ are the absorbances at 0 and 10 min. Each sample was measured in triplicate (*n* = 3).

### 2.8 Fourier Transform Infrared Spectroscopy

FTIR spectra were recorded using a spectrophotometer (IRPrestige‐21, Shimadzu, Kyoto, Japan) equipped with an attenuated total reflectance (ATR) accessory with a diamond crystal. Analyses were performed in the spectral range of 4000–400 cm^−1^, with a resolution of 4 cm^−1^ and 32 accumulated scans per sample. The objective was to associate the main peaks with the predominant chemical bonds present in each molecule, enabling the identification of the primary bands of the protein hydrolysate according to the significant mathematical model (hydrolysate obtained using alcalase at a concentration of 1.1025% and hydrolysis time of 4.615 h).

Approximately 3 mg of lyophilized protein hydrolysate was mixed with 100 mg of potassium bromide (KBr, ≥99% purity, Sigma‐Aldrich, St. Louis, MO, USA). Baseline correction was performed using the instrument software, and spectra normalization was carried out in OriginPro 2024 (OriginLab Corporation, Northampton, MA, USA).

### 2.9 Statistical Analysis

All experimental data were analyzed using analysis of variance (ANOVA), and significant differences between means were determined by Tukey's test (*p* < 0.05). The relationship between solubility and DH was further explored through principal component analysis (PCA).

A central composite rotatable design (CCRD) was employed to determine the optimal hydrolysis conditions for the protein concentrate and to evaluate the emulsifying capacity of hydrolysates produced using alcalase and papain. RSM was used to generate contour plots, and a complete quadratic model was fitted to describe the enzymatic process for each enzyme. Optimization used a desirability function to maximize solubility, EAI, and ESI within the experimental domain.

All statistical analyses and graphical representations were performed using OriginPro 2024 (OriginLab Corporation, Northampton, MA, USA) and Statistica 13.5 (TIBCO Software Inc., Palo Alto, CA, USA).

## 3 Results

### 3.1 Physicochemical Characterization of the Protein Concentrate

The moisture content of the protein concentrate obtained from Nile tilapia, dried in an air circulation oven at 50°C for 18 h, was 13.52%. This value is higher than that reported by Silva (2003) for fish soup prepared from mechanically separated black piranha meat (10.53%), dried under similar conditions at 60°C for 18 h. In contrast, Vidal et al. (2011) reported moisture contents ranging from 0.37% to 2.11% for protein concentrates from Nile tilapia filleting residues dried at 65°C for 18 h, indicating that drying temperature and raw material markedly affect final moisture levels. Studies applying alkaline solubilization‐isoelectric precipitation to fish proteins have reported residual moisture contents typically between approximately 8% and 11% (Nolsøe and Undeland 2002; Pires et al. 2017), values closer to those obtained in the present study.

The lipid content of the protein concentrate was 7.34%, similar to the 6.08% reported by Souza et al. (2022) in Nile tilapia mortadella formulated with different proportions of frozen MSM. Lipid levels in fish protein concentrates are affected by both the lipid content of the starting material and the efficiency of lipid‐removal steps during processing. Inadequate lipid removal can compromise the oxidative stability of the concentrate during storage, as lipids, especially polyunsaturated fatty acids, are prone to peroxidation (Shahidi and Ambigaipalan 2018). This factor must be considered in downstream applications, particularly when formulating products with extended shelf life.

The protein content of the concentrate was 65.94%, a value comparable to that reported by Vidal‐Campello et al. (2021) (62.39%) in the industrial‐scale production of tilapia protein concentrates from filleting residues. Romadhoni et al. (2016) similarly reported a protein content of 63.78% for *Ophicephalus striatus* protein concentrates. According to Balaswamy et al. (2007), the protein content in concentrates is influenced by several factors, including fish species, extraction methods, solvents, drying techniques, and the composition of the raw material. Moisture content also plays a key role, as there is an inverse relationship between protein and water content (Ogawa 1999). The effective water removal achieved in this study likely contributed to the high protein concentration. In addition, the mild processing conditions may have helped to preserve protein integrity, preventing excessive denaturation and aggregation that could otherwise reduce solubility and functionality.

The DH results for alcalase and papain are presented in Table 2. The highest DH for alcalase (9.50%) was observed in FPH8 (0.75% enzyme, 4.615 h), whereas for papain, the maximum value (13.15%) occurred in FPH4 (1.0% enzyme, 4.0 h). These values are consistent with the literature, which reports that milder alcalase conditions tend to yield moderate DH levels suitable for maintaining desirable functional properties. J. A. Pedroza Filho et al. (2021) applied alcalase to MSM from tilapia, obtaining DH values between 6% and 10% under similar pH and temperature conditions. In contrast, Shu et al. (2018) reported DH values of around 12% for alcalase applied to tilapia by‐products for 2 h at 50°C.

Differences in DH between enzymes can be attributed to their catalytic mechanisms and substrate specificities. Alcalase, a serine endopeptidase from *B. licheniformis*, preferentially cleaves peptide bonds on the carboxyl side of hydrophobic amino acids (Kristinsson and Rasco 2000). In contrast, papain, a cysteine protease from *C. papaya*, exhibits broader specificity, with activity toward bonds involving hydrophobic or bulky aromatic residues, and retains stability at slightly acidic to neutral pH (Tavano 2013). This broader specificity may explain the higher DH values observed with papain across most treatments, as also reported by Yarnpakdee and Kristinsson (2012) for hydrolysates obtained from fish viscera.

For alcalase, extending hydrolysis time did not necessarily lead to proportional increases in DH under all conditions. For instance, FPH1 (0.5%, 1 h) reached 2.34%, whereas FPH2 (0.5%, 4 h) increased to 7.80%, but similar trends were not consistently observed at other enzyme–substrate combinations. This behavior suggests that substrate depletion, product inhibition, or time‐dependent conformational changes may limit further proteolysis (Nelson and Cox 2014). Conversely, papain generally showed a clearer positive relationship between time and DH at the same enzyme concentration, as observed for FPH1–FPH2 and FPH3–FPH4. This contrast highlights the importance of tailoring hydrolysis parameters to the specific catalytic profile of each enzyme.

The DH values obtained here also align with those reported for other fish species. Dong et al. (2008) found a DH of 23% using alcalase (0.5%, pH 8.0, 60°C) for 6 h on Pacific whiting, while Vázquez et al. (2017) reported a wide DH range (5.6%–29.3%) for *Scyliorhinus canicula* discards, depending on pH and temperature. Such variability underscores the substantial influence of reaction conditions and substrate characteristics on proteolysis outcomes.

Despite the relatively long hydrolysis time and enzyme dosage, the DH values remained moderate, which is consistent with previous reports indicating that extensive proteolysis can reach a plateau due to substrate depletion, limited accessibility of peptide bonds, and possible product inhibition (Najafian et al. 2013; Noman et al. 2018).

Table 3 shows the solubility of FPH as a function of pH for both enzymes. For alcalase‐treated samples, solubility was generally higher at alkaline pH, with FPH1 reaching 31.94% at pH 9.0. The lowest solubility for alcalase occurred near pH 5.0, close to the pI of myofibrillar proteins, where net charge is minimal and protein–protein interactions dominate, leading to aggregation (De santis et al. 2022).

For papain hydrolysates, solubility was consistently lower than for alcalase across most pH values, despite higher DH. This apparent paradox can be explained by the nature of peptides generated: papain's extensive hydrolysis may produce more hydrophobic peptides prone to aggregation, thus reducing solubility (Chalamaiah et al. 2010). The highest solubility of papain was observed in FPH5 at pH 5.0 (8.60%), suggesting that specific hydrolysis patterns can enhance solubility even near the pI under certain conditions.

The solubility patterns observed here are consistent with reports by Chen et al. (2015), who found that myofibrillar protein solubility is influenced by ionic strength and pH, with higher solubility at alkaline pH due to increased electrostatic repulsion. Similarly, Raghavan and Kristinsson (2008) reported that hydrolysis generally improves solubility by exposing polar amino acid residues; however, excessive cleavage can lead to small peptides with higher hydrophobicity, thereby decreasing their water dispersibility.

Processing conditions, such as temperature, shear, ionic strength, and drying method, can also significantly affect protein solubility (Grossmann and McClements 2023). The alkaline solubility advantage of alcalase hydrolysates in this study suggests potential for their use in food systems requiring high solubility, such as protein beverages or emulsions.

Overall, the results show that enzyme type, concentration, and hydrolysis time had marked effects on both DH and solubility. Alcalase hydrolysates exhibited better solubility profiles, particularly at neutral to alkaline pH levels, making them promising for applications where dispersibility is crucial. Papain hydrolysates, while exhibiting higher DH, may require further processing (e.g., blending with other hydrolysates or using selective fractionation) to optimize functionality for specific food products.

These findings are relevant for the valorization of Nile tilapia by‐products, contributing to circular economy strategies in the aquaculture sector. By selecting appropriate hydrolysis conditions, it is possible to tailor the functional properties of protein hydrolysates for different applications, ranging from sports nutrition supplements to emulsifying agents in processed foods.

Given that both DH and solubility varied considerably depending on enzyme type, concentration, and pH, a multivariate approach was applied to better capture the interrelationships among these variables. PCA was therefore employed to simultaneously evaluate DH and solubility at different pH values for all treatments. This method allows the identification of patterns and correlations that may not be evident in univariate analyses, highlighting how specific combinations of processing parameters influence the overall functional profile of the hydrolysates.

The DH increased with both enzyme concentration and reaction time for all tested conditions. This behavior reflects the progressive cleavage of peptide bonds as enzymatic exposure and catalytic time are extended, consistent with kinetic patterns reported for FPHs (Chalamaiah et al. 2010; Tran et al. 2023).

Alcalase, an endopeptidase with broad specificity toward hydrophobic residues, exhibited a more controlled increase in DH, whereas papain, which combines endo‐ and exopeptidase activity, promoted faster cleavage and higher DH values at equivalent concentrations.

However, elevated DH does not necessarily translate into improved functional performance. Once the peptide chains become excessively short, most of the nitrogen becomes TCA‐soluble, and the DH value reaches an apparent plateau, even though further scission continues to occur (Baek and Cadwallader 1995).

This saturation explains why the maximum experimental DH (13.80%) at 1.1025% alcalase for 4.615 h was not disproportionately higher than intermediate points. Similar trends have been observed in tilapia hydrolysates, where extensive cleavage results in diminished interfacial behavior due to the loss of amphiphilic balance (Kumari et al. 2023). The results of the PCA are presented in the following section.

### 3.2 Principal Component Analysis

The highest solubility was observed at pH 5.0 for sample FPH5, an FPH produced using papain under moderate hydrolysis conditions. In contrast, the lowest solubility values were found for sample FPH6 across all tested pH values (5.0, 7.0, and 9.0), with statistically similar low values also observed for FPH4 at pH 9.0. This indicates that, for papain hydrolysates, specific combinations of enzyme concentration and hydrolysis time can markedly reduce water dispersibility.

For hydrolysates produced with alcalase, an inverse relationship between solubility and the DH was evident: as DH decreased, solubility tended to increase. This trend suggests that maintaining partial protein integrity may favor intermolecular interactions with water, thus enhancing solubility. Conversely, for papain hydrolysates, the relationship was reversed: solubility decreased as DH increased. This could be linked to the generation of short peptides with greater hydrophobic character, which are less prone to water dispersion, as discussed by Chalamaiah et al. (2010) and corroborated by our solubility data.

**FIGURE 1:**
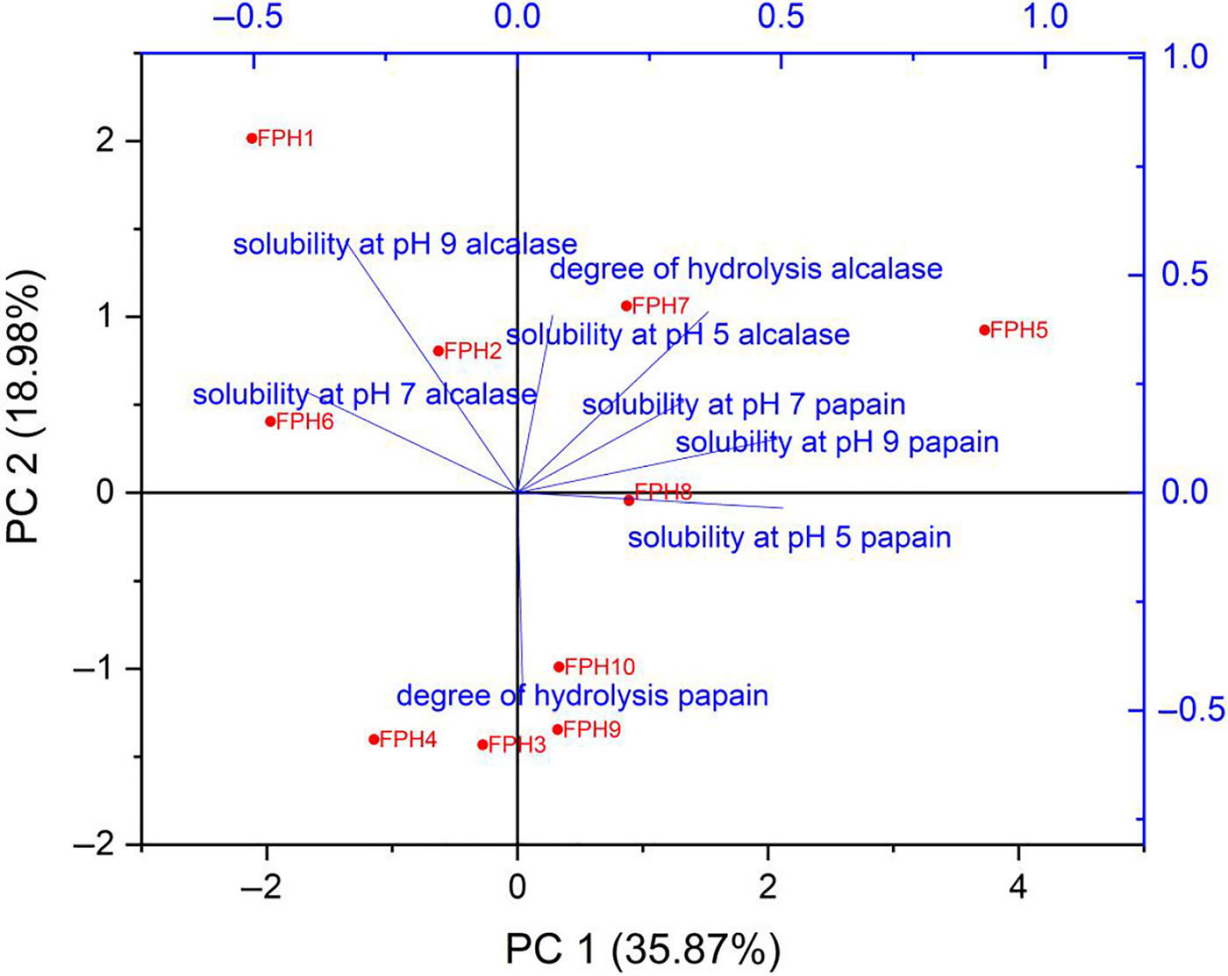
Biplot graph of PCA for the analysis of the degree of hydrolysis and solubility for the enzymes alcalase and papain.

The PCA biplot (Figure 1) illustrates these relationships, showing that the projection of PC1 × PC2 (where PC1 and PC2 stand for principal component 1 and principal component 2, respectively) explained 54.85% of the total variance. PC1, which accounted for 35.87% of the variance, correlated positively with solubility at pH 7.0–9.0 and with emulsifying activity (EAI) and stability (ESI), and positively discriminated samples treated with alcalase at higher concentrations and longer times, which clustered in the quadrant of high solubility and high emulsifying indices. PC2, explaining 18.98% of the variance, was more closely associated with DH values and helped separate samples obtained with papain, which tended to shift toward higher DH but lower solubility and functional scores. This multivariate pattern confirms that enzyme specificity and hydrolysis degree jointly define the techno‐functional profile of FPHs.

These findings align with the enzymatic specificity patterns reported by Najafian (2013), who observed that hydrolysis of *Pangasius sutchi* myofibrillar proteins using papain and alcalase resulted in increasing DH for both enzymes with longer hydrolysis times, although papain consistently reached higher DH values than alcalase. This difference arises from their distinct cleavage preferences: alcalase, a serine endoprotease, preferentially cleaves at the C‐terminal of hydrophobic amino acids (Kristinsson and Rasco 2000; Zhang et al. 2022), while papain, a cysteine protease, exhibits broader substrate specificity and can generate a higher number of low‐molecular‐weight peptides in shorter times.

Structural aspects of the hydrolysates also play a role in the PCA distribution. As noted by Shu et al. (2018), variations in amino acid sequence, peptide chain length, and surface hydrophobicity influence both solubility and functional properties. Moreover, Liu et al. (2015) reported that differences in hydrophobic group exposure and secondary structure, detectable through techniques such as absorption spectroscopy, directly affect protein–water interactions and thereby influence PCA clustering patterns.

Overall, the PCA confirms that enzyme type, concentration, and hydrolysis time not only determine DH but also modulate solubility and emulsifying behavior in distinct ways, resulting in clear multivariate separation of treatments. These insights reinforce the potential of PCA as a valuable statistical tool to visualize complex relationships between physicochemical and techno‐functional parameters in protein hydrolysates, complementing univariate analyses and aiding in the selection of optimal processing conditions for targeted functionalities.

### 3.3 Emulsifying Capacity (EAI and ESI)

FPH is a surfactant material containing hydrophilic and hydrophobic groups that promote the formation of an oil‐in‐water emulsion. Protein capacity can be measured by its ability to form and stabilize emulsions, providing the unit area of the interface stabilized per unit protein weight (m^2^ g^−1^) and its continuity over time through the EAI and ESI (Noman et al. 2018).

Regarding the emulsifying capacity determined in this study for the hydrolysates obtained through the action of alcalase and papain enzymes at pH 5.0, 7.0, and 9.0, the mean EAI and ESI values are presented in Table 4.

The emulsion turbidity was evaluated at a wavelength of 500 nm. Regarding emulsifying activity (EAI), measured in m^2^ g^−1^, the highest value was observed in the alcalase sample FPH6, with an enzyme concentration of 1.1025%. This sample showed statistically similar mean values for hydrolysis at pH 5.0 (3.57E + 00 ± 1.67E‐01 m^2^ g^−1^) and pH 9.0 (3.20E + 00 ± 2.03E‐01 m^2^ g^−1^). For papain, the emulsifying activity values were lower than those observed for alcalase, with the highest EAI values concentrated at pH 9.0 for the samples FPH2, FPH3, and FPH9. The emulsifying activity was affected by pH, with significant differences observed across various pH levels.

Concerning emulsion stability (ESI), expressed in min, the highest value was recorded for sample FPH3 (90.34 ± 2.98 min) in alcalase at pH 7.0, and for samples FPH6 (914.15 ± 42.38 min) and FPH8 (909.95 ± 34.42 min) in papain, also at pH 7.0. The lowest ESI values were found in alcalase samples FPH5 (12.09 ± 0.11 min), FPH6 (11.84 ± 0.02 min), FPH9 (12.23 ± 0.32 min), and FPH10 (11.93 ± 0.18 min) at pH 5.0, with statistically similar means. For papain, the lowest ESI value was observed in sample FPH10 (9.15 ± 0.21 min) at pH 5.0, with statistically similar values at pH 7.0 and 9.0.

According to Kumari et al. (2023), polypeptides unfold in alkaline pH due to their negative charges, enhancing interfacial orientation and effectively exposing hydrophilic and hydrophobic residues, which promotes significant interactions at the oil–water interface and increases the EAI of protein hydrolysates. On the other hand, the stability of the absorbed layer covering the fat globule and its interaction with the hydrophobic component are key factors for ESI (Vo et al. 2020).

**FIGURE 2:**
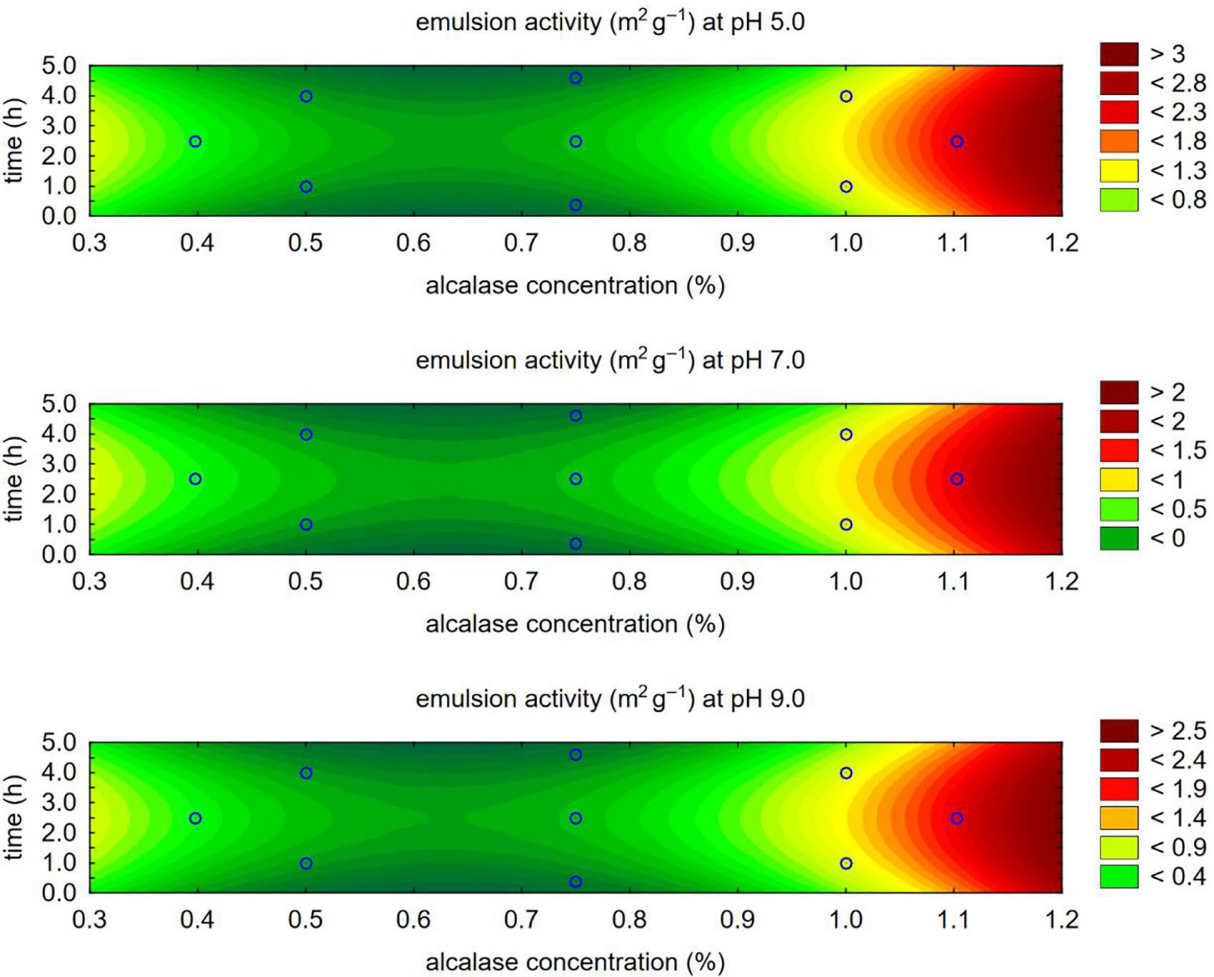
Contour plot for emulsifying activity with alcalase enzyme.

Analyzing the emulsifying capacity of alcalase individually concerning the concentration (%) and hydrolysis time (h) determined via CCRD (Table 1), it was found that the emulsifying activity (EAI) at pH 5.0, 7.0, and 9.0 achieved its highest values in the region corresponding to a hydrolysis time of approximately 2.5 h and higher enzyme concentrations (Figure 2).

**TABLE 1:** Experimental design matrix for enzymatic hydrolysis of protein concentrate from Nile tilapia, showing coded and decoded values for the factors analyzed.

Experiments	Alcalase concentration (%), *x*_1_	Time (h), *x*_2_
FPH1	0.5 (−1)	1 (−1)
FPH2	0.5 (−1)	4 (1)
FPH3	1.0 (1)	1 (−1)
FPH4	1.0 (1)	4 (1)
FPH5	0.3975 (−1.41)	2.5 (0)
FPH6	1.1025 (1.41)	2.5 (0)
FPH7	0.75 (0)	0.385 (−1.41)
FPH8	0.75 (0)	4.615 (1.41)
FPH9 (C)	0.75 (0)	2.5 (0)
FPH10 (C)	0.75 (0)	2.5 (0)

Experiments	Papain concentration (%), *x*_1_	Time (h), *x*_2_
FPH1	0.5 (−1)	1 (−1)
FPH2	0.5 (−1)	4 (1)
FPH3	1.0 (1)	1 (−1)
FPH4	1.0 (1)	4 (1)
FPH5	0.3975 (−1.41)	2.5 (0)
FPH6	1.1025 (1.41)	2.5 (0)
FPH7	0.75 (0)	0.385 (−1.41)
FPH8	0.75 (0)	4.615 (1.41)
FPH9 (C)	0.75 (0)	2.5 (0)
FPH10 (C)	0.75 (0)	2.5 (0)

**TABLE 2:** Means and standard deviations for the degree of enzymatic hydrolysis in Nile tilapia protein concentrate as a function of enzyme concentration and time for alcalase and papain.

Experiments	Enzyme concentration (%)	Time (h)	Degree of hydrolysis (%)	
			Alcalase	Papain
FPH1	0.5	1	2.34^e^ ± 0.39	8.84^e^ ± 0.14
FPH2	0.5	4	7.80^b^ ± 0.12	10.74^d^ ± 0.03
FPH3	1	1	1.27^f^ ± 0.10	10.59^d^ ± 0.24
FPH4	1	4	4.60^d^ ± 0.12	13.15^a^ ± 0.19
FPH5	0.3975	2.5	5.66^c^ ± 0.05	11.40^c^ ± 0.66
FPH6	1.1025	2.5	3.24^d^ ± 0.03	12.14^b^ ± 0.66
FPH7	0.75	0.385	4.09^d^ ± 0.18	8.34^e^ ± 0.13
FPH8	0.75	4.615	9.50^a^ ± 0.91	12.16^b^ ± 0.11
FPH9	0.75	2.5	1.26^f^ ± 0.02	11.35^c^ ± 0.18
FPH10	0.75	2.5	2.86^e^ ± 0.09	10.74^cd^ ± 0.50

*Note*: Means in the same column, followed by different lowercase letters, differ significantly according to Tukey's test at the 5% significance level.

**TABLE 3:** Means and standard deviations of the solubility of fish protein hydrolysates as a function of pH.

	Solubility (%)		
	Alcalase		
Experiments	**pH 5**	**pH 7**	**pH 9**
FPH 1	7.94^cC^ ± 0.27	15.12^aB^ ± 0.35	31.94^aA^ ± 0.76
FPH 2	11.83^aB^ ± 0.36	11.53^cB^ ± 0.23	22.65^bA^ ± 0.27
FPH 3	7.13^dC^ ± 0.18	10.67^dB^ ± 0.22	14.45^dA^ ± 0.51
FPH 4	8.29^cC^ ± 0.27	12.64^bB^ ± 0.37	17.09^cA^ ± 0.79
FPH 5	10.31^bC^ ± 0.18	11.07^cB^ ± 0.18	14.05^dA^ ± 0.43
FPH 6	11.53^aB^ ± 0.32	16.08^aA^ ± 0.46	16.99^cA^ ± 0.35
FPH 7	9.81^bB^ ± 0.27	9.81^eB^ ± 0.27	16.88^cA^ ± 0.25
FPH 8	10.31^bC^ ± 0.26	12.03^bB^ ± 0.18	15.01^dA^ ± 0.41
FPH 9	8.09^cB^ ± 0.22	11.58^cA^ ± 0.13	11.73^eA^ ± 0.10
FPH 10	7.73^cB^ ± 0.15	11.53^cA^ ± 0.11	11.63^eA^ ± 0.13

**Experiments**	**Papain**		
	**pH 5**	**pH 7**	**pH 9**
FPH 1	4.39^dB^ ± 0.17	5.43^cA^ ± 0.75	4.68^dB^ ± 0.04
FPH 2	5.06^cB^ ± 0.01	5.73^bA^ ± 0.65	4.78^dB^ ± 0.03
FPH 3	5.17^cB^ ± 0.02	4.33^dC^ ± 0.32	5.57^bA^ ± 0.03
FPH 4	4.95^cB^ ± 0.13	5.73^bA^ ± 1.30	4.10^eC^ ± 0.03
FPH 5	8.60^aA^ ± 0.01	6.33^aC^ ± 0.75	6.81^aB^ ± 0.04
FPH 6	4.27^dA^ ± 0.13	4.27^dA^ ± 0.65	4.27^eA^ ± 0.01
FPH 7	5.06^cB^ ± 0.01	5.17^cB^ ± 0.65	5.63^bA^ ± 0.19
FPH 8	4.57^dC^ ± 0.26	5.96^aB^ ± 0.65	6.80^aA^ ± 0.10
FPH 9	6.07^bA^ ± 0.01	5.47^cB^ ± 0.38	4.98^cC^ ± 0.08
FPH 10	5.92^bA^ ± 0.01	4.91^cB^ ± 1.35	5.02^cB^ ± 0.10

*Note*: Means in the same column, followed by different lowercase letters, differ according to Tukey's test at a 5% significance level, comparing solubility at each pH across all samples. Means in the same row, followed by different uppercase letters, differ according to Tukey's test at a 5% significance level, comparing solubility variations across different pH values for each sample.

**FIGURE 3:**
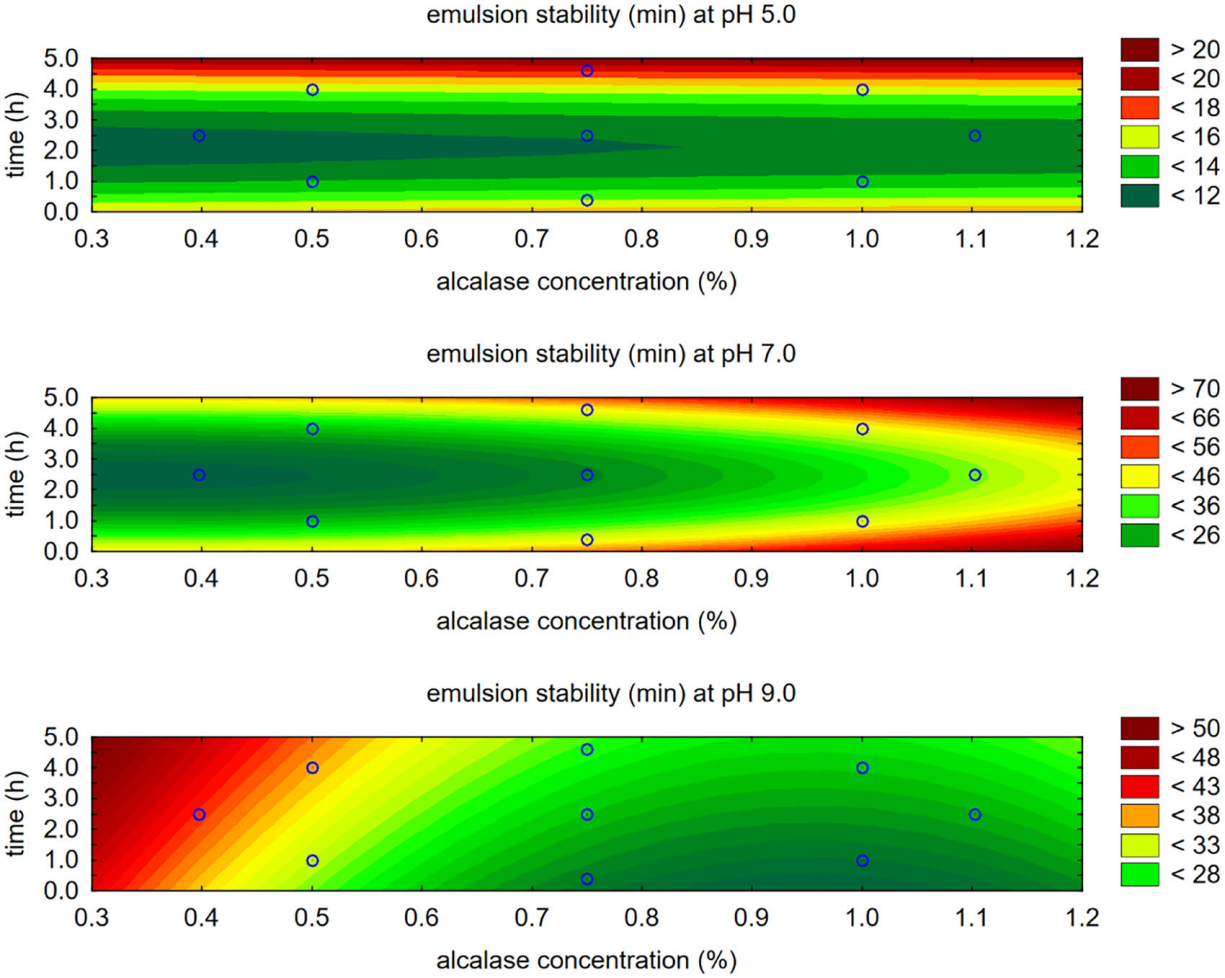
Contour plot for emulsion stability with alcalase enzyme.

For emulsion stability (ESI), also evaluated at pH levels 5.0, 7.0, and 9.0, the contour plot (Figure 3) shows somewhat divergent regions regarding alcalase concentration and hydrolysis time. While the trend generally indicates higher concentration and longer hydrolysis times, except at pH 9.0, the desirability function was applied to determine a mathematical model for the optimal combination of these two variables, which influence the emulsifying capacity as well as the solubility achieved with alcalase.

Certain functional properties of protein hydrolysates play a predominant role, as they determine the main characteristics of the final product and its potential applications. Thus, the origin of the protein used as raw material to produce protein hydrolysates, along with the DH achieved, must be controlled in line with the key functional properties to be explored (Centenaro et al. 2009).

According to Panyam and Kilara (1996), extensive protein hydrolysis results in a significant loss of emulsifying properties, which decrease linearly with the DH. Larger peptides promote greater emulsion stability, while the presence of smaller peptides impairs emulsion formation and stability. Emulsifying properties also depend on the initial solubility of the protein.

The more dissolved the protein is within the emulsion system, the more effective the interface between the oil phase and the continuous phase can be during emulsification. However, forming a cohesive and elastic interfacial film through the adsorption of protein molecules at the interface can be hindered by the predominance of small peptides. This may explain the decrease in emulsifying capacity as the DH increases. In this study, the FPH6 experiment with alcalase yielded significantly better emulsifying activity (EAI) at acidic (pH 5.0), neutral (pH 7.0), and alkaline (pH 9.0) conditions.

For emulsion stability results, the FPH3 experiment with alcalase exhibited the longest activity time (90.34 min) at neutral pH (7.0). Both experiments exhibited low DHs, with FPH6 (DH = 3.24%) and FPH3 (DH = 1.27%), which corroborates the literature findings discussed above.

**FIGURE 4:**
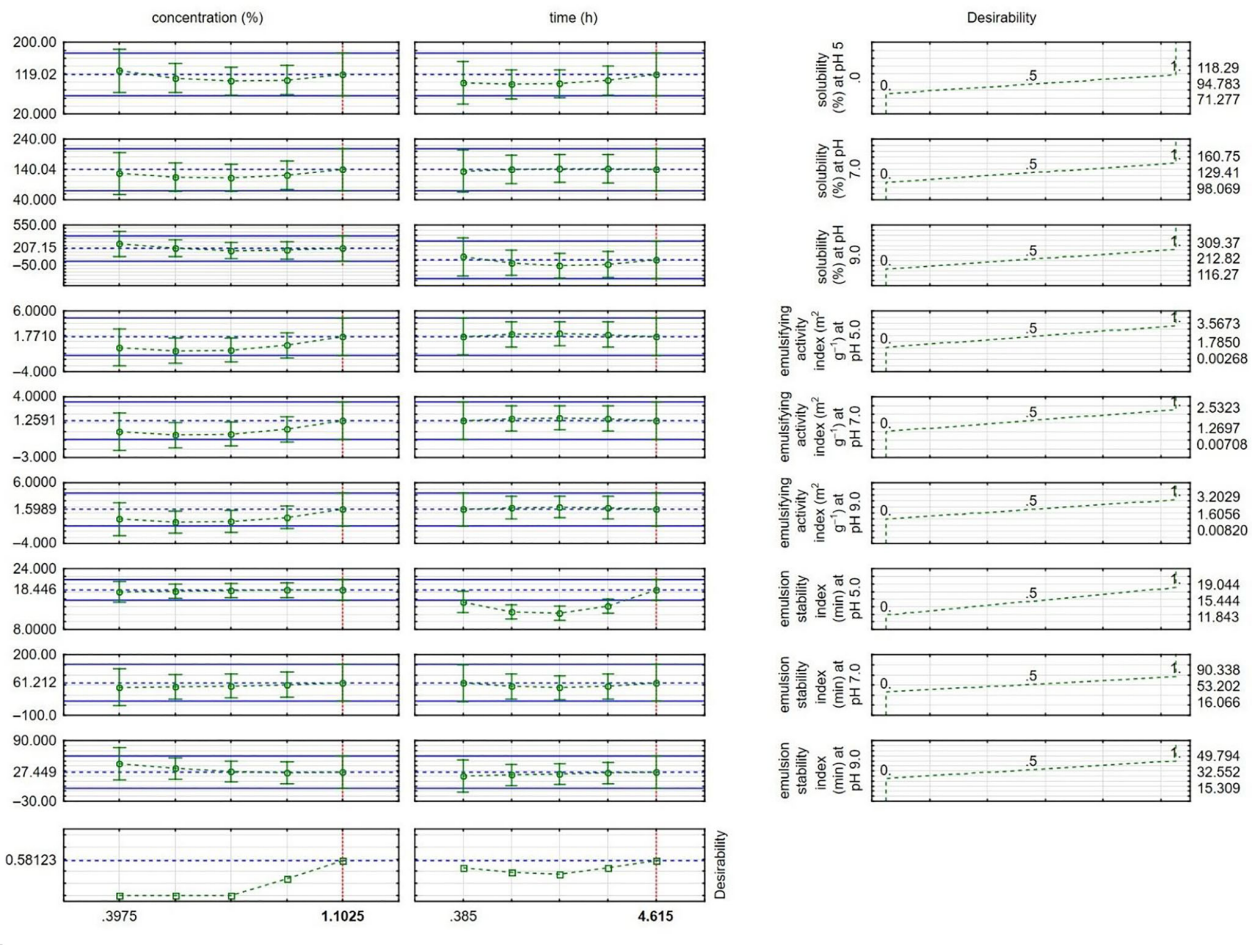
Graphical representation of the desirability function considering concentration and time for solubility and emulsifying capacity of the alcalase enzyme.

As a graphical result of the desirability function, Figure 4 presents the optimized response for the combination of alcalase concentration and hydrolysis time. It indicates that the ideal condition would be an alcalase concentration of 1.1025%, combined with a hydrolysis time of 4.615 h.

**FIGURE 5:**
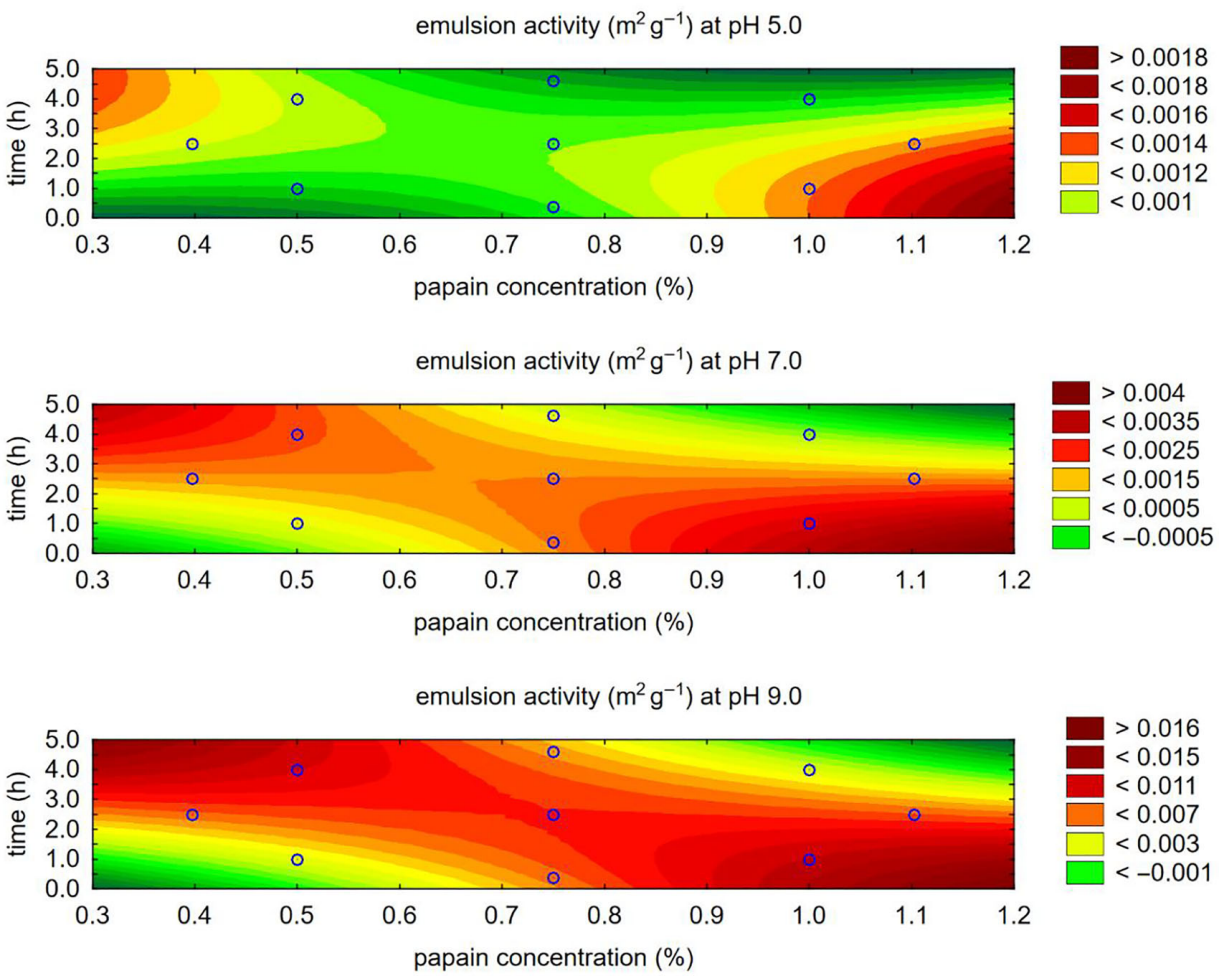
Contour plot for emulsion activity with papain enzyme.

Analyzing individually the emulsifying capacity of papain concerning concentration (%) and time (h) determined via CCRD (Table 1), it is observed that the emulsion activity, at pH 5.0, 7.0, and 9.0, shows higher values located in the region with lower time and lower concentration (Figure 5).

**FIGURE 6:**
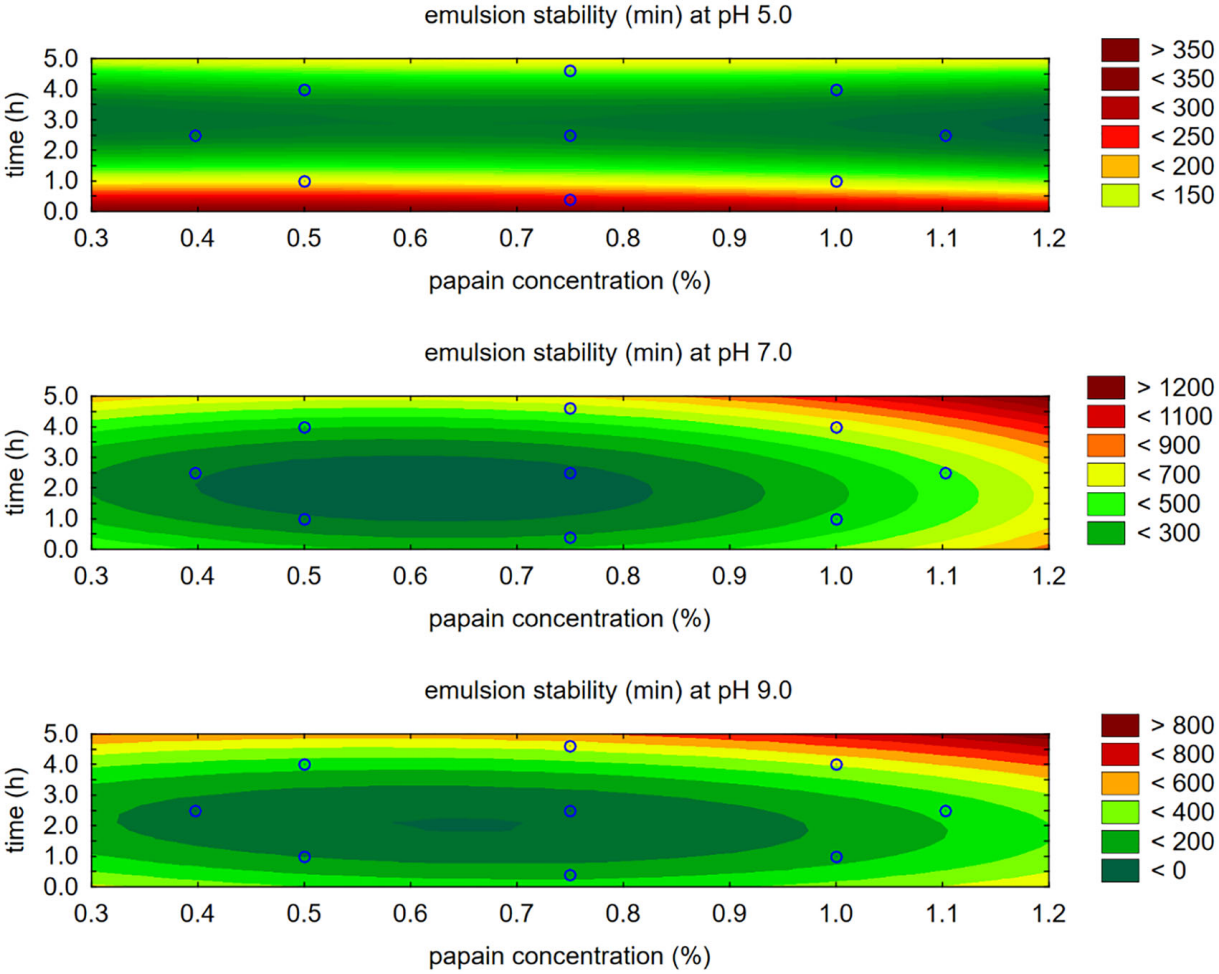
Contour plot for emulsion stability with papain enzyme.

For emulsion stability, also observed at pH 5.0, 7.0, and 9.0, the contour plot (Figure 6) shows somewhat divergent regions regarding the concentration of papain and time. However, this study observed lower emulsion stability values for papain compared to alcalase (Table 4). While higher concentrations and longer times are generally preferred, except at pH 5.0, the desirability function was used to determine the optimal combination of these two variables that determines emulsifying capacity and solubility, as determined by papain.

**TABLE 4:** Means and standard deviations for the fish protein hydrolysate samples studied, considering the emulsifying capacity of both enzymes.

	Emulsifying activity index (EAI) (m^2^ g^−1^)			Emulsion stability index (ESI) (min)		
	Alcalase			Alcalase		
Experiments	pH 5	pH 7	pH 9	pH 5	pH 7	pH 9
FPH 1	1.12E‐02^bB^ ± 2.68E‐04	1.09E‐02^bA^ ± 1.41E‐03	1.05E‐02^bB^ ± 4.32E‐04	13.29^cC^ ± 0.11	20.67^eB^ ± 1.25	28.54^cA^ ± 0.73
FPH 2	1.20E‐02^bC^ ± 1.57E‐04	1.18E‐02^bA^ ± 1.01E‐03	8.30E‐03^cB^ ± 7.51E‐04	14.67^bC^ ± 0.04	30.02^cB^ ± 0.84	49.79^aA^ ± 3.93
FPH 3	2.06E‐02^bA^ ± 8.99E‐04	2.75E‐02^bA^ ± 3.49E‐04	4.10E‐02^bA^ ± 4.83E‐04	14.54^bB^ ± 0.13	90.34^aA^ ± 2.98	15.31^eB^ ± 0.24
FPH 4	1.43E‐02^bB^ ± 2.51E‐04	2.58E‐02^bA^ ± 2.28E‐03	1.38E‐02^bA^ ± 1.61E‐03	15.19^bC^ ± 0.46	19.52^eB^ ± 1.31	33.29^bA^ ± 3.10
FPH 5	1.14E‐02^bB^ ± 1.21E‐04	1.79E‐02^bB^ ± 1.26E‐04	8.20E‐03^cA^ ± 5.93E‐04	12.09^dC^ ± 0.11	20.08^eB^ ± 0.24	35.53^bA^ ± 2.81
FPH 6	3.57E+00^aA^ ± 1.67E‐01	2.53E+00^aB^ ± 2.03E‐01	3.20E+00^aA^ ± 9.98E‐02	11.84^dB^ ± 0.02	22.63^dA^ ± 0.54	22.66^dA^ ± 0.85
FPH 7	2.61E‐02^bB^ ± 1.03E‐04	2.73E‐02^bA^ ± 3.42E‐03	3.23E‐02^bA^ ± 1.09E‐03	14.07^bC^ ± 0.17	16.07^fB^ ± 1.19	28.09^cA^ ± 1.02
FPH 8	2.68E‐03^cB^ ± 1.95E‐04	7.08E‐03^cA^ ± 1.91E‐03	3.29E‐02^bA^ ± 2.88E‐03	19.04^aB^ ± 1.31	47.41^bA^ ± 1.12	15.33^eC^ ± 0.29
FPH 9	1.61E‐02^bA^ ± 1.71E‐04	3.12E‐02^bB^ ± 7.67E‐05	3.69E‐02^bA^ ± 6.66E‐04	12.23^dB^ ± 0.32	25.61^dA^ ± 0.20	27.32^cA^ ± 3.16
FPH 10	1.68E‐02^bA^ ± 7.98E‐04	2.00E‐02^bA^ ± 6.34E‐04	1.65E‐02^bA^ ± 8.61E‐04	11.93^dC^ ± 0.18	17.40^fB^ ± 0.67	22.71^dA^ ± 0.80

	Emulsifying activity index (EAI) (m^2^ g^−^ **^1^)**			Emulsion stability index (ESI) (min)		
	Papain			Papain		
Experiments	pH 5	pH 7	pH 9	pH 5	pH 7	pH 9
FPH 1	1.33E‐03^aA^ ± 1.51E‐04	1.52E‐03^cA^ ± 1.54E‐04	5.61E‐03^bA^ ± 1.73E‐04	51.89^eC^ ± 4.50	138.89^dA^ ± 10.37	109.47^eB^ ± 3.41
FPH 2	1.26E‐03^aC^ ± 3.25E‐05	2.37E‐03^bB^ ± 1.63E‐04	1.38E‐02^aA^ ± 1.69E‐03	57.31^eB^ ± 4.27	151.73^cA^ ± 5.16	45.18^gC^ ± 3.05
FPH 3	1.77E‐03^aB^ ± 1.53E‐04	4.56E‐03^aB^ ± 1.27E‐04	1.65E‐02^aA^ ± 3.32E‐03	37.79^fB^ ± 1.21	50.16^eA^ ± 3.81	28.77^hC^ ± 3.98
FPH 4	8.23E‐04^bC^ ± 1.62E‐05	1.26E‐03^cB^ ± 9.65E‐05	8.24E‐03^bA^ ± 2.54E‐04	65.28^dB^ ± 5.93	150.43^cA^ ± 5.64	60.74^fB^ ± 0.78
FPH 5	5.22E‐04^bB^ ± 1.62E‐05	1.30E‐03^cA^ ± 6.05E‐05	3.83E‐03^cA^ ± 1.97E‐04	85.00^cB^ ± 2.90	118.26^dA^ ± 5.64	129.32^dA^ ± 6.71
FPH 6	9.52E‐04^bA^ ± 3.74E‐05	1.72E‐04^dB^ ± 6.14E‐06	7.98E‐04^dA^ ± 3.42E‐05	54.69^eC^ ± 0.98	914.15^aA^ ± 42.38	503.28^bB^ ± 46.35
FPH 7	1.60E‐04^cB^ ± 1.23E‐05	5.10E‐04^dB^ ± 1.62E‐05	2.40E‐03^cA^ ± 2.22E‐04	399.33^aA^ ± 30.68	307.49^bB^ ± 7.92	187.69^cC^ ± 15.88
FPH 8	4.48E‐04^bA^ ± 1.23E‐05	1.54E‐04^dB^ ± 6.14E‐06	4.79E‐04^dA^ ± 4.64E‐05	101.02^bC^ ± 3.84	909.95^aA^ ± 34.42	774.11^aB^ ± 74.87
FPH 9	1.14E‐03^aB^ ± 7.08E‐05	1.34E‐03^cB^ ± 7.67E‐05	1.36E‐02^aA^ ± 9.73E‐04	41.40^fB^ ± 3.00	128.04^dA^ ± 8.12	27.24^hC^ ± 1.97
FPH 10	6.51E‐04^bB^ ± 1.62E‐05	2.21E‐03^bA^ ± 8.49E‐04	2.75E‐03^cB^ ± 5.53E‐05	9.15^gA^ ± 0.21	13.80^fA^ ± 4.59	14.12^iA^ ± 0.37

*Note*: Means in the same column, followed by distinct lowercase letters, differ significantly according to Tukey's test at a 5% significance level for the DH and solubility at each pH when comparing all samples. Means in the same row, followed by distinct uppercase letters, differ significantly according to Tukey's test at a 5% significance level for solubility variation across different pH values for each sample.

Abbreviation: FPH, fish protein hydrolysate.

According to Duarte et al. (1998), the effect of pH on emulsion stability is complex, as proteins can form more cohesive and viscous interfacial films near their pI, which is beneficial for emulsion stability. This could explain the behavior observed with the papain hydrolysate (FPH) at pH 5.0, considered the pI for animal proteins.

**FIGURE 7:**
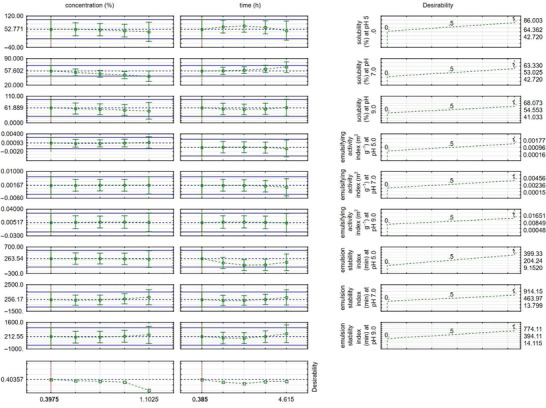
Graphical representation of the desirability function considering concentration and time for solubility and emulsifying capacity of the papain enzyme.

As a result of the desirability function, the optimized response for the combination of papain concentration and time is illustrated in Figure 7. It can be observed that the ideal concentration would be 0.3975% papain, combined with a hydrolysis time of 0.385 h.

The behavior of the enzymes in the contour surface and desirability graphs shows good solubility, activity, and emulsion stability. Therefore, the choice of enzyme for hydrolysis conditions depends on the desired product profile.

The quadratic models fitted to the experimental data demonstrated satisfactory adequacy, with high coefficients of determination (*R* ^2 ^> 0.90) and no significant lack of fit (*p* > 0.05).

RSM efficiently described the combined effects of enzyme concentration and time, enabling identification of the optimal region. The desirability function approach indicated that the best global response corresponded to alcalase 1.1025% for 4.615 h, producing DH = 13.80% and simultaneous increases in solubility, EAI, and ESI compared with the design‐center condition (DH = 2.06%).

Weighting functional properties more heavily than DH itself allowed for the prioritization of practical performance over extensive cleavage. This strategy aligns with current trends in functional protein ingredient design, emphasizing techno‐functional efficiency over maximal hydrolysis yield.

In the study by Wasswa et al. (2007), protein hydrolysates from grass carp skin were obtained through enzymatic hydrolysis using alcalase. The hydrolysis was carried out using the pH‐stat method, and the hydrolysates were analyzed for functional properties. It was noticeable that the functional properties of FPHs can be modified according to the DH. The emulsifying capacity of the hydrolysates decreased as protein hydrolysis increased. An inverse relationship was observed between DH and emulsifying capacity, possibly due to the presence of smaller peptides, which are less effective in stabilizing emulsions.

Functional properties influence the utility of an ingredient in food and govern physical behavior during preparation, processing, and storage. Solubility is one of the most important properties of proteins and protein hydrolysates. Many other functional properties, such as emulsification and foam formation, are affected by solubility. In another study by Balti et al. (2011), the hydrolyzed protein concentrates showed minimum solubility at pH 4.0, which may correspond to the isoelectric point of protein hydrolysates, and high solubility at alkaline pH. In this study, the lowest pH tested was 5.0, and the highest was 9.0. In alcalase experiments, solubility was lower at pH 5.0, with higher values observed at pH 7.0 and 9.0. The pH 5.0, 7.0, and 9.0 experiments showed similar solubility values for papain.

### 3.4 Optimization of Enzymatic Hydrolysis Conditions

Now, comparing the optimized enzyme sample, with a concentration of 1.1025% alcalase and a time of 4.615 h, with the sample considering the central point of concentration and time, the following results are presented in Table 5 for the DH.

**TABLE 5:** Averages and deviations for the degree of hydrolysis as a function of the alcalase enzyme concentration and time.

Experiments	Degree of hydrolysis (%)
OFPH	13.80^a ^± 0.02
CPFPH	2.06^b ^± 0.36

*Note*: Averages in the same column, followed by distinct lowercase letters, differ by Tukey's test at the 5% significance level for the degree of hydrolysis and for solubility at each pH, comparing all samples. Abbreviations: CPFPH, fish protein hydrolysate at the central point; OFPH, optimized fish protein hydrolysate.

With a significant difference (*p* < 0.05), the DH was higher (13.80 ± 0.02%) for the optimized sample compared to the central point sample (2.06 ± 0.36%). As previously discussed, even moderate variations in the DH can markedly influence functional properties. Depending on the type of peptides generated, higher DH values may lead to structural changes that negatively affect solubility, emulsifying activity, and other properties. Pires et al. (2015) emphasize that comparing results between studies is difficult because hydrolysis conditions are not standardized. Moreover, determining DH using different methods generally does not allow for comparable results.

According to Fogaça (2018), a considerable amount of peptide bonds is hydrolyzed at the beginning of the reaction. After a specific time, this amount decreases until a constant behavior is observed. For solubility, the values for the optimized sample increased compared to the central point sample, with significant differences in solubility at pH 5.0 and 7.0 (Table 6). At a pH of 9.0, the samples showed no significant differences. It is essential to note that the highest solubility was observed at pH 7.0 for the optimized sample, as illustrated more clearly in Figure 4.

**TABLE 6:** Averages and deviations for the samples considering the solubility of the alcalase enzyme.

Experiments	Solubility (%)		
	pH 5	pH 7	pH 9
OFPH	9.30^a ^± 0.66	18.47^a ^± 2.27	12.22^a ^± 1.27
CPFPH	7.91^b ^± 0.14	11.65^b ^± 1.07	11.68^a ^± 0.78

*Note*: Averages in the same column, followed by distinct lowercase letters, differ from each other according to Tukey's test at the 5% significance level for the degree of hydrolysis and for solubility at each pH, comparing all samples with each other.

Abbreviations: CPFPH, fish protein hydrolysate at the central point; OFPH, optimized fish protein hydrolysate.

**FIGURE 8:**
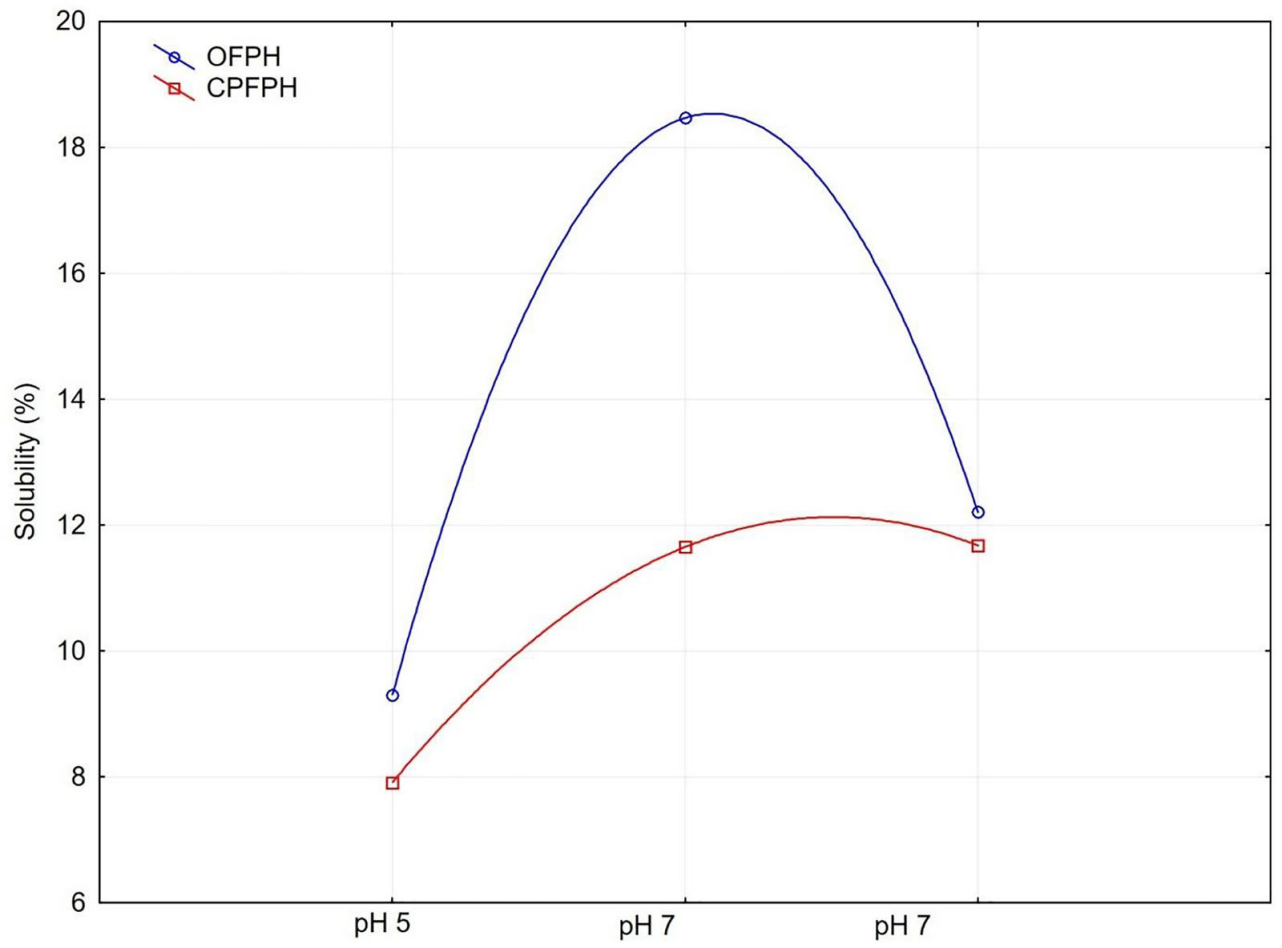
Development of solubility.

Regarding the properties of protein hydrolysates, solubility is among the most important (Figure 8), as low solubility can result in an unappealing appearance in the final product (Liu et al. 2015). In the present study, the optimized sample exhibited its highest solubility at pH 5.0, indicating that under acidic conditions, the peptides generated may have structures more favorable for dispersion in aqueous media.

Again, it is worth noting that this water‐washing procedure is species‐dependent. When using the washing and dilution procedure with water, most MPs from fish species, such as cod, can be solubilized in water. Large volumes of water are required to disperse the proteins at neutral pH. Attention must also be paid to the ionic strength, which must be low enough to achieve solubilization. However, while the extraction of MPs with low ionic strength can help separate different MPs, their application in food technology may be limited, as the ionic strength of food cannot be adjusted to such a low value. No study has been published on the functional properties of water‐soluble MPs prepared with this procedure (Chen et al. 2017).

The reduced solubility observed at pH 5.0 is similar to that reported by Marasca (2020), who obtained a protein isolate from weakfish (*Cynoscion guatacupa*) and found lower solubility results at pH 5.5. This pH value is close to the pI of proteins (Panyam and Kilara 1996; Mullally et al. 1994; Kristinsson and Hultin 2003), where electrostatic charges tend to neutralize intramolecularly, resulting in lower interaction with the solvent. Another study using protein concentrates from croaker, obtained through chemical solubilization of proteins, also showed reduced solubility near the pI but high solubility at pH values above 7.0, with greater dispersion in alkaline conditions (Fontana et al. 2009).

Since protein solubility is closely related to their structural states, it is often used to measure the extent of denaturation during extraction, isolation, and purification processes. Therefore, solubility is one of the most significant functional properties of protein hydrolysates (Damodaran et al. 2007). The peptides released by the alcalase hydrolysis in this study showed higher solubility at pH 7.0 (neutral), followed by a decrease in solubility at pH 9.0, the opposite result found in the study by Benelhadj et al. (2016) for protein isolate from microalgae (*Spirulina platensis*), which showed higher solubility at pH 10.0. In hydrolysates of Asian swamp eel (*Monopterus* sp.) treated with alcalase enzyme, Halim and Sarbon (2019) found higher solubility at a pH of 10.0. However, no significant differences were observed between the other pH values (4.0 and 7.0) evaluated. pH values influence the solubility properties of FPHs, as the peptide bonds can be cleaved over a wide pH range. Thus, solubility is affected by variations in pH.

Solubility is also related to protein particle size, meaning that smaller particles have a greater surface area contact, which increases the propensity for solubility and optimizes activities such as foam and emulsion formation. Hydrophobic interactions promote protein–protein interactions, decreasing solubility, while ionic interactions promote protein‐water interactions, increasing solubility (Abuine et al. 2019).

The better solubility of the hydrolysate is due to the smaller size of its molecules compared to the intact protein and the ionizable amino and carboxyl groups of the newly exposed amino acids, which increase the hydrophilicity of the hydrolysate. Although increased solubility has a positive relationship with the extent of hydrolysis, care must be taken to avoid over‐hydrolyzing the protein (Mullally et al. 1994). This was likely the case in our present study, where papain enzyme hydrolysis showed lower solubility results as the DH increased.

Protein solubility profiles revealed a strong dependence on pH and enzyme type. Solubility decreased markedly near the isoelectric point (pI ≈ 5.0) because of minimal net charge and aggregation of protein molecules, as expected for myofibrillar proteins (Kristinsson and Hultin 2003).

Alcalase hydrolysates exhibited higher solubility at neutral and alkaline pH levels, likely due to the increased exposure of polar groups and reduced molecular weight, which enhances hydration.

In contrast, papain hydrolysates presented slightly lower solubility at pH 5–7, indicating that their peptide distribution and terminal residues favored hydrophobic aggregation under these conditions.

These results align with previous reports, which show that the solubility of fish hydrolysates is highly influenced by enzyme specificity and hydrolysis conditions (Zu et al. 2023).

From a practical perspective, the improved solubility at pH ≥7 suggests a potential application of optimized hydrolysates in neutral or slightly basic food systems, such as protein beverages or emulsion sauces.

The results of emulsion activity and its stability are shown in Table 7.

**TABLE 7:** Averages and deviations for the samples considering the emulsion activity and stability of the alcalase enzyme.

Experiments	Emulsifying activity index (EAI) (m^2^ g^−1^)		
	pH 5	pH 7	pH 9
OFPH	1.08E‐02^b^ ± 1.84E‐04	1.42E‐02^b^ ± 3.22E‐04	7.35E‐03^b^ ± 3.10E‐05
CPFPH	1.60E‐02^a^ ± 3.99E‐04	2.56E‐02^a^ ± 2.31E‐04	2.67E‐02^a^ ± 4.57E‐04

**Experiments**	**Emulsion stability index (ESI)** **(min)**		
	**pH 5**	**pH 7**	**pH 9**
OFPH	17.57^a^ ± 0.14	23.45^a^ ± 0.35	28.71^a^ ± 0.06
CPFPH	12.08^b^ ± 0.18	21.51^b^ ± 0.67	25.01^a^ ± 1.79

*Note*: Averages in the same column, followed by distinct lowercase letters, differ from each other according to Tukey's test at the 5% significance level for the degree of hydrolysis and for solubility at each pH, comparing all samples with each other.

Abbreviations: CPFPH, fish protein hydrolysate at the central point; OFPH, optimized fish protein hydrolysate.

In terms of emulsion stability (min), higher values were observed for the optimized sample, without showing a significant difference (*p* < 0.05) at pH levels of 5.0 and 7.0. As the pH increased, the emulsion stability value also increased. The opposite occurred for emulsion activity. Higher values were observed for the central point sample compared to the optimized sample, with the highest value (2.67E‐02 ± 4.57E‐04) found at pH 9.0 for the CFPH sample.

The EAI and ESI followed patterns consistent with solubility results. Higher EAI and ESI values were obtained for alcalase hydrolysates under optimized conditions, confirming their superior interfacial activity.

The moderate hydrolysis promoted by alcalase appears to generate peptides with an optimal amphiphilic balance, where hydrophobic segments enable adsorption to the oil–water interface and polar regions stabilize the surrounding aqueous phase.

Over‐hydrolysis caused by papain can lead to the formation of very small peptides that diffuse rapidly but fail to form cohesive interfacial films, resulting in lower EAI and ESI despite higher DH.

This trade‐off between DH and emulsifying performance was also reported by Singh et al. (2023) and Park et al. (2023), emphasizing that controlled proteolysis is crucial to retain functional integrity.

**FIGURE 9:**
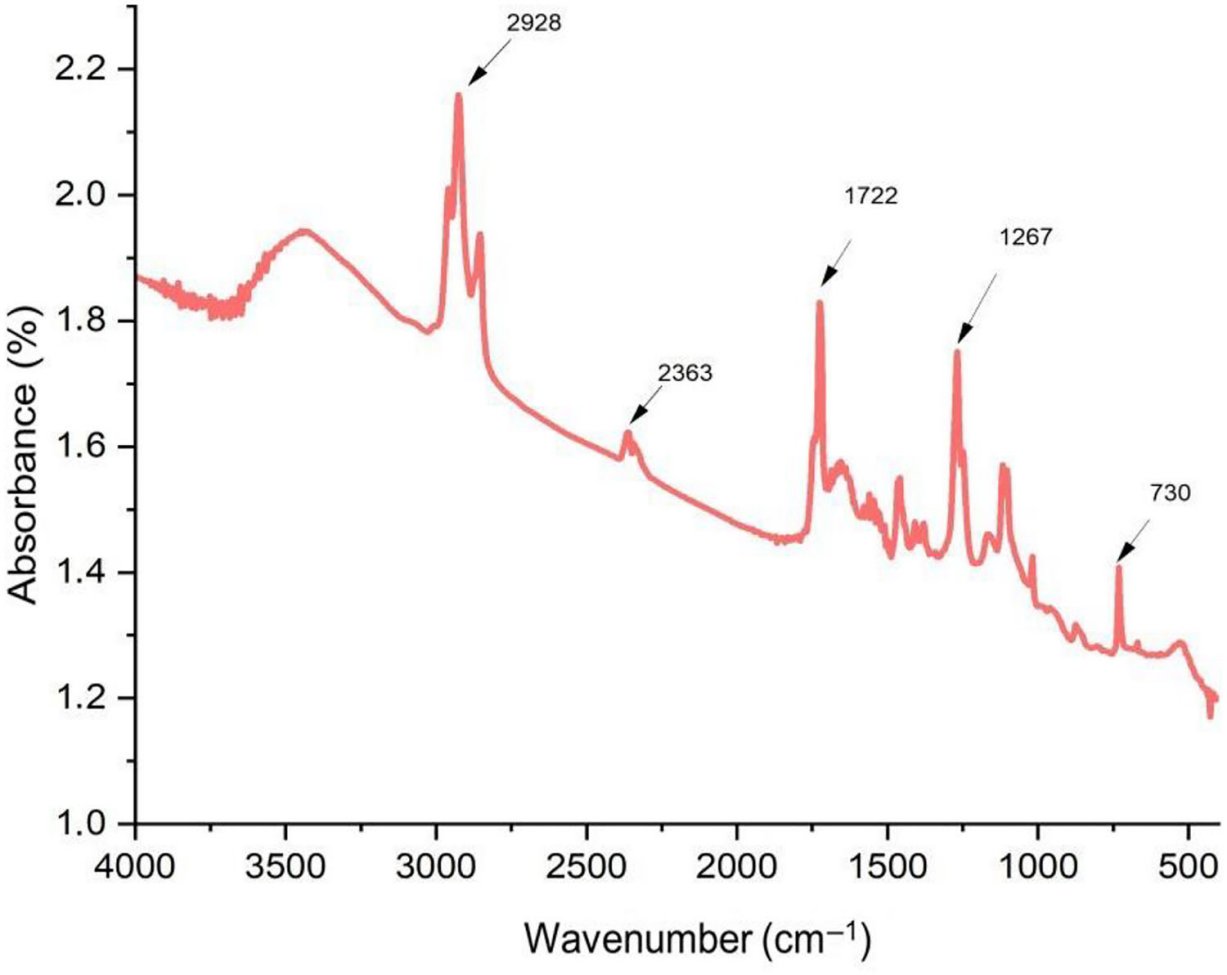
FTIR spectra for the enzymatically hydrolyzed samples optimized by alcalase enzyme.

The optimized FPHs therefore exhibit potential as natural emulsifiers in clean‐label formulations, reducing reliance on synthetic surfactants. Figure 9 shows the FTIR spectroscopy spectra for the optimized alcalase enzyme sample.

The chemical structure of a molecule is the primary factor that determines its vibrational frequencies, influenced by bond forces and atomic masses. FTIR analysis qualitatively characterizes the reduction of the protein chain. The protonation state of most side chains is reflected in the spectrum (Böcker et al. 2017; Barth 2007). Various structural features of proteins are linked to peptide bonds, such as functional performance, proton transport, protein synthesis, information exchange, and other functions associated with peptide amide binding (Ji et al. 2020).

In this study, FTIR analysis was performed only on the hydrolysate obtained under the optimized condition with alcalase (1.1025%, 4.615 h). FTIR analysis was not conducted on the original protein concentrate due to constraints on sample availability and the exploratory nature of this work. Therefore, the spectra are interpreted qualitatively to identify the main amide bands and infer general structural rearrangements rather than to provide a complete before‐and‐after comparison of the hydrolysis process. Future studies from our group will include FTIR and complementary structural analyses (e.g., peptide size distribution, amino acid composition, and zeta potential) for both unhydrolyzed and hydrolyzed samples to obtain a more detailed picture of the enzymatic modifications. For better data perception, only the average spectra of the samples are shown in Figure 9. When enzymatic hydrolysis occurs, in addition to intrinsic changes in the protein's primary structure, it also promotes changes in secondary structures, which can be observed in the FTIR absorption spectrum.

Regarding the main bands of the FTIR spectrum for the optimized sample, five bands can be observed at positions 2928, 2363, 1722, 1267, and 730 cm^−1^. The peak at 2928 cm^−1^ represents the stretching modes of the methyl and methylene groups, resulting in a symmetric stretching vibration at approximately 2872 cm^−1^ and an asymmetric stretching vibration at approximately 2962 cm^−1^. These are attributed to the hydrophobic amino acid side chains (Alancay et al. 2023). The amide I band (1700–1600 cm^−1^) is commonly used to assess the secondary structure of proteins (Garcia‐Sifuentes et al. 2021; Yousefi et al. 2017). In this study, the band observed at 1722 cm^−1^ can be attributed to the stretching vibration of the carbonyl (C═O) group, which is associated with the presence of polar groups (Ahmed et al. 2018; Yousefi et al. 2017). This feature can enhance the solubility of a molecule in polar solvents through dipole–dipole interactions, such as with water, and is consistent with the significant increase in solubility observed for the optimized hydrolysate (OFPH), as shown in Table 6 and Figure 8.

Silva et al. (2015) found similar absorption bands for gelatins from the skins of corvina and bijupirá at 1773 and 1723 cm^−1^, respectively. The amide III band (1267 cm^−1^) results from bending the single H bond and stretching the ─N bond with C─H and N─H deformation vibrations (Glassford et al. 2013).

A study with ora‐pro‐nobis (OPN) leaves, an edible plant with a high protein value, presented bands with similar values to those found in this study: 3430 cm^−1^ (O─H stretch bonded to H, or N─H stretch), 2923 cm^−1^ (aldehyde stretch), 1750 cm^−1^ (aldehyde/ketone ─C═O stretch), 1644.1 cm^−1^ (C═C stretch), 1519.6 cm^−1^ (NH bending), 1384.0 cm^−1^ (CH_3_ bending), 1251.9 cm^−1^ (C─H bending in plane), and 1156.0 cm^−1^ (C─N amine stretch) (Maciel et al. 2021).

Acid‐soluble collagen (ASC) and pepsin‐soluble collagen (PSC) obtained from mixed by‐products of different fish species were extracted and evaluated. The functional group amide A was located at 3293 and 3288 cm^−1^ for ASC and PSC, respectively, indicating the presence of amino (NH) groups involved in hydrogen bond associations, with a strong hydrogen bond in both ASC and PSC. The functional group amide B is associated with the asymmetric stretching vibrations of ═CH and ─NH^+3^ bonds. The amide B functional groups in ASC and PSC were detected at 2932 and 2933 cm^−1^, respectively (Garcia‐Sifuentes et al. 2021).

The band at 730 cm^−1^ can be attributed to phosphate groups (PO_4_^3−^), found in fish spines (Nawaz et al. 2020), and to the bending vibrations of amino acid groups (CH_2_), highly intense bonds that are not exposed to solvent water, with defined interactions with the protein environment (Barth 2007).

The ATR‐FTIR spectra of the optimized hydrolysate (alcalase 1.1025%, 4.615 h) showed characteristic amide I (≈1650 cm^−1^) and amide II (≈1540 cm^−1^) bands with noticeable shifts compared to the non‐hydrolyzed concentrate, suggesting modification of the secondary structure.

The reduction in α‐helix signals and the relative increase in β‐sheet content indicate partial unfolding and rearrangement of the protein backbone, consistent with the enhanced solubility and interfacial activity. Because FTIR was applied only to the optimized condition, the analysis was qualitative; future work will include spectra of the untreated concentrate to monitor structural evolution during hydrolysis. Comparable FTIR patterns were observed by Zu et al. (2023) for the enzymatic hydrolysis of silver carp scales, confirming that similar molecular transitions underpin functionality across aquatic protein matrices.

## 4 Conclusion

The protein concentrate obtained from MSM of Nile tilapia exhibited a proximate composition consistent with previous reports for fish by‐products, characterized by a high protein content, moderate lipid levels, and low moisture, indicating efficient separation and confirming its suitability as a raw material for the development of functional protein ingredients. This contributes to the valorization of MSM, a widely available yet underutilized co‐product of fish processing, within a circular‐economy framework.

Enzymatic hydrolysis with alcalase and papain generated FPHs with distinct DHs and techno‐functional profiles. Under the conditions evaluated, papain generally produced higher DH values, whereas alcalase led to hydrolysates with superior solubility and emulsifying properties. This behavior reflects the different catalytic mechanisms and substrate specificities of the two proteases: papain, with broader specificity, tends to generate a higher proportion of low‐molecular‐weight peptides, while alcalase promotes a more moderate breakdown that preserves peptide lengths compatible with interfacial film formation. The data also showed that increasing hydrolysis time and enzyme concentration do not necessarily produce proportional gains in DH, suggesting the occurrence of substrate depletion, product inhibition, and structural constraints that limit further proteolysis.

Hydrolysis conditions significantly affected protein solubility across the entire pH range tested. All hydrolysates exhibited higher solubility at alkaline pH, whereas reduced solubility around pH 5.0 was associated with the vicinity of the isoelectric point, contributing to greater variability among treatments. For papain, higher DH was often accompanied by reduced solubility, indicating that the predominance of short, hydrophobic peptides can compromise dispersion in aqueous media. In contrast, alcalase hydrolysates with intermediate DH values tended to show a more favorable balance between exposed hydrophilic and hydrophobic residues, resulting in higher solubility and better emulsifying activity (EAI) and stability (ESI).

Multivariate analysis reinforced these trends. PCA clearly separated treatments according to enzyme type, hydrolysis severity, and functional performance, with alcalase treatments at higher concentrations and longer times clustering in the region of higher solubility and emulsifying indices, while papain treatments shifted toward higher DH but lower functional scores. This multivariate pattern confirms that enzyme selection and process conditions jointly define the techno‐functional “fingerprint” of the resulting hydrolysates, highlighting PCA as a valuable tool to guide process tuning beyond univariate comparisons.

RSM identified hydrolysis conditions capable of simultaneously maximizing DH and functional properties. For papain, moderate conditions resulted in hydrolysates with acceptable solubility and DH, while the optimized condition for alcalase (1.1025% enzyme, 4.615 h) produced a satisfactory DH, high solubility at neutral pH, and the highest emulsion stability values among the treatments. These findings suggest that controlled proteolysis with alcalase is particularly promising for producing FPHs with emulsifying potential, making them suitable for use as clean‐label stabilizers or protein‐enrichment agents in food formulations.

FTIR analysis of the optimized alcalase hydrolysate revealed characteristic amide bands and spectral features consistent with partial unfolding and rearrangement of the protein backbone, in line with the observed improvements in solubility and interfacial properties. Although FTIR was applied only to the optimized condition in this exploratory study, the results support the notion that structural rearrangements at the molecular level underpin the functional changes induced by enzymatic hydrolysis.

Overall, this study demonstrates that Nile tilapia MSM is a viable substrate for producing functional protein hydrolysates and that the rational selection of enzyme type, concentration, and reaction time is crucial to tailor DH, solubility, and emulsifying behavior. Future work should incorporate complementary structural analyses (peptide size distribution, amino acid composition, and zeta potential), as well as validation in real food matrices and sensory studies, to refine structure–function relationships and consolidate the industrial applicability of these FPHs in clean‐label and sustainability‐oriented product development.

## Author Contributions

**Poliana dos Santos Mendes**: conceptualization, investigation, methodology, validation, project administration. **Flávia Aparecida Reitz Cardoso**: methodology, validation, writing – review and editing, writing – original draft. **Maria Fernanda Giugiolli**: methodology, validation. **Gabriela Wolhmuth**: methodology, validation. **Maria Isabel Campos Oliveira**: methodology, validation. **Vitória de Freitas Fante**: methodology, validation. **Jéssica Novelo Nascimento Brenag**: methodology, validation. **Evandro Bona**: methodology, validation. **Patrícia Casarin**: methodology, validation. **Adriana Aparecida Droval Arcain**: conceptualization, methodology, validation, writing – review and editing, writing – original draft, supervision.

## Funding

Author Flávia Aparecida Reitz Cardoso has received research support from Conselho Nacional de Desenvolvimento Científico e Tecnológico—Brasil (CNPq) (Grant numbers [302078/2023‐1]).

## Conflicts of Interest

The authors declare no conflicts of interest.
